# Latent TB Infection Diagnosis in Population Exposed to TB Subjects in Close and Poor Ventilated High TB Endemic Zone in India

**DOI:** 10.1371/journal.pone.0089524

**Published:** 2014-03-10

**Authors:** Rajpal S. Kashyap, Amit R. Nayak, Hari M. Gaherwar, Aliabbas A. Husain, Seema D. Shekhawat, Ruchika K. Jain, Milind S. Panchbhai, Dhananjay V. Raje, Hemant J. Purohit, Girdhar M. Taori, Hatim F. Daginawala

**Affiliations:** 1 Biochemistry Research Laboratory, Central India Institute of Medical Sciences, Nagpur, Maharashtra, India; 2 MDS Bio-Analytics Pvt. Ltd, Nagpur, Maharashtra, India; 3 Environmental Genomic Unit, National Environmental Engineering Research Institute, Nagpur, Maharashtra, India; Fundació Institut d'Investigació en Ciències de la Salut Germans Trias i Pujol. Universitat Autònoma de Barcelona. CIBERES, Spain

## Abstract

**Background:**

The present study was designed to investigate the utility of Quantiferon TB gold (QFT-G) and Tuberculin skin test (TST) for diagnosis of latent TB infection (LTBI) in high crowding TB endemic zone of Nagpur, India and their comparison with associated risk factors.

**Methods:**

Out of 342 eligible participants, QFT-G and TST were performed in 162 participants.

**Results:**

The prevalence of LTBI observed according to QFT-G and TST was 48% and 42% respectively, with an agreement of 52.47%. QFT-G positivity was associated with age while TST positivity was associated with body mass index (BMI). Duration of exposure emerged as a key risk factor significantly associated with both the tests.

**Conclusion:**

The prevalence of LTBI was quite high in the studied zone as detected by both the evaluated tests and thus, the combination of both the tests will be best predictive for LTBI in such high TB endemic region**s**.

## Introduction

Tuberculosis remains one of the major causes of mortality and morbidity worldwide [1], [2] Household contact with active tuberculosis (TB) patients has been implicated as one of the crucial factors responsible for transmission of TB disease [3], [4]. The risk of TB infection increases even after a short period of exposure with the infected person in families with high household crowding index denoted by the number of co-residents per room, and living in low socio-economic and poor hygienic conditions. Despite the prevalence of latent tuberculosis infection (LTBI) in India, limited measures are undertaken for its diagnosis in densely populated TB endemic regions. Thus, the identification of LTBI in family members living in such regions at an early stage is required to prevent the progression to active disease and minimize the incidence of TB infection in India.

For decades, the diagnosis of LTBI has relied on the Tuberculin Skin Test (TST) [5]. Unfortunately, the TST suffers from a number of limitations, the most serious being false-positive responses due to cross reactivity caused either by infection with non-tuberculous mycobacteria (NTM), or by bacillus Calmette-Guerin (BCG) vaccination [6], [7].During past few years, Quantiferon-TB Gold test (QFT-G), an *in-vitro* whole-blood interferon (IFN-γ) assay based on *Mycobacterium tuberculosis* (MTB) specific antigens [i.e early secreted antigenic target 6 (ESAT-6) and culture filtrate protein 10 (CFP-10),] has been extensively evaluated for TB diagnosis. The number of published reports clearly indicates that QFT-G is more reliable marker than TST with higher sensitivity as well as specificity and a better correlation with exposure to MTB [8], [9]

The comparative diagnostic utility of both QFT-G and TST in healthcare workers, certain risk groups and high TB endemic countries have been published [10]. To the best of our knowledge, no study has been carried out among the family members of active TB patients living in close and poor ventilated high TB endemic zone in India. Therefore, the present study was designed to investigate the diagnostic utility of QFT-G and TST for LTBI in such TB endemic region of Nagpur, India and their comparison with associated risk factors. In addition, follow up study was conducted with the aim to study impact of risk factors in participants positive for both the evaluated tests in initial study.

## Materials and Methods

### Ethics statement

The study was approved by the Ethical Committee of Central India Institute of Medical Sciences, (CIIMS), Nagpur. All clinical investigations were conducted according to the principles expressed in the declaration of Helsinki. All the participants were given oral explanation of the study, as well as written consents were taken from all the recruited participants. Since the study was also conducted on children, a written informed consent was obtained prior to inclusion from the immediate caretaker, or next of kin on behalf of children participating in the study.

### Study design

The present study was conducted in a high TB endemic area, in a specific locality of Nagpur district in Maharashtra, India. The area has high household crowding index, with an average of six to eight members living in small closed rooms. Poor ventilation (houses in close vicinity with one window per house) unhygienic living conditions, use of common toiletries, and poor socio-economic status were among the other risk factors associated with the participants in this region.

A total of 545 participants were enrolled from this region during the camps organized between May 2009 and June 2012. The enrolled participants were screened for eligibility using a set of pre-specified inclusion and exclusion criteria. In brief, participants were recruited from the families who had at least one sputum positive pulmonary tuberculosis patient (PTB) living in the same house hold, for at least 2 months before the start of anti TB medication, who have high probability of repeated exposure and who are at high risk of infection. The identification of TB patients and their family details were obtained from patient's records available in TB DOTS centre located in the vicinity of the study area.

Detailed information about each participant was obtained through a structured questionnaire to arrive at other possible risk factors of LTBI, along with the exposure with TB patient. Base line characteristics such as age, gender, weight, height, occupation, education, symptoms, behavioral factors, duration of exposure and contact type with active TB patients were recorded. Other information's like any prior TST, presence of underlying illnesses and infections experienced in the last three months were also recorded. BCG vaccination status was recorded based on the examination of BCG scar on left forearm. Participants (mostly females) who refused to show BCG scar on forearm due to community ethics, were questioned orally regarding the same.

A total of 203 participants were excluded from the study based on their refusal to give blood. The rest 342 participants who matched the inclusion criteria were selected for the study. Amongst them, the participants who had earlier history of TB or previously been treated with anti TB treatment (ATT) (n = 58), pregnant women (n = 12), the participants who migrated to other places (n = 66) and children with age younger than 10 years (n = 22) (to avoid impact of BCG reactivity on TST) were futher excluded from the study. The exposure period of the participants were characterized as moderate based on living with active TB patients for two to three months and high for those living for more than three months.14 participants with exposure of less than a month were not included in the study while 8 participants in which either QFT-G and/or TST were not performed were also excluded from the study.

For follow up studies, participants were identified form earlier records available with us and also with help of local physician in the study area. Based on brief counseling; we were able to recruit 14 participants. Among them 2 participants failed to come during blood collection and 4 more participants were further excluded because they refused to give blood at the time of the study. In the remaining 8 follow up cases, TST was not done in 2 cases.

### QuantiFERON-TB Gold test (QFT-G) and Tuberculin skin test (TST)

QFT-G (Cellestis Limited, Carnegie, Victoria, Australia) test was conducted according to the manufacturer's instructions. Blood was directly collected into two tubes one containing only heparin as negative control, and the second tube containing heparin with overlapping peptides sequences of CFP-10 and ESAT-6, and TB7.7. After incubation for 16–24 hours plasma was removed and frozen until used. The IFN-γ values were calculated by subtracting the value of negative control and the cut-off value was found to be 0.35 IU/mL.

The TST was administered intradermally using the Mantoux technique. 5 TU PPD RT23 (Staten Serum Institute, Denmark) as recommended by India's Revised National Tuberculosis Control Programme (RNTCP), and the diameter of induration was read after 48–72 hours by an experienced and certified tuberculin reader. An induration of at least 10 mm was considered positive. For comparison, 5 mm and 15 mm indurations were also evaluated.

### Statistical analysis

Descriptive statistic like frequency (percentage) was obtained for demographic, behavioral and clinical factors measured on nominal scale. The test (QFT-G/TST) result was treated as an outcome, and the proportion of positive cases corresponding to different levels of factors (covariates) was obtained. Unadjusted odds ratio was obtained as a measure of impact of each factor on the test outcome. Factors showing significant influence in bivariate analysis were included in the multivariable logistic regression model resulting into adjusted odds ratio estimates for relevant factors. Age and sex were retained in the model being important biological covariates irrespective of their statistical significance. BCG was not included in the model due to lack of confirmation on 45 participants. A subgroup analysis was performed with confirmed BCG cases in the multivariate model (n = 117) [File S1]. A parsimonious regression model was derived using Wald's criterion for variable selection. The resulting model fitness was evaluated by referring to Hosmer-Lemeshow test. The analysis was performed independently for the two test outcomes and the associations of factors with the outcomes in the final models were compared.

Percent agreement and Cohen's kappa statistic with 95% confidence interval were obtained to determine the extent of concordance between the tests. In addition, the above statistics were obtained according to the levels of factors to determine if there existed any trend of agreement across the levels of factors. Moreover, the association between the factors and combination of test outcomes were assessed using multinomial univariate logistic regression. The concordant negative (QFT-G−/TST−) was treated as reference outcome, and the other three joint outcomes i.e., concordant positive (QFT-G+/TST+), discordant QFT-G+/TST− and discordant QFT-G−/TST+ were compared against the reference. Odds ratios along with 95% confidence intervals were obtained for each factor and the model fitness was evaluated using log-likelihood ratio test.

All the analysis was performed using *R*-2.15.1 programming language with pre-validated programs.

## Results

**Figure 1:**
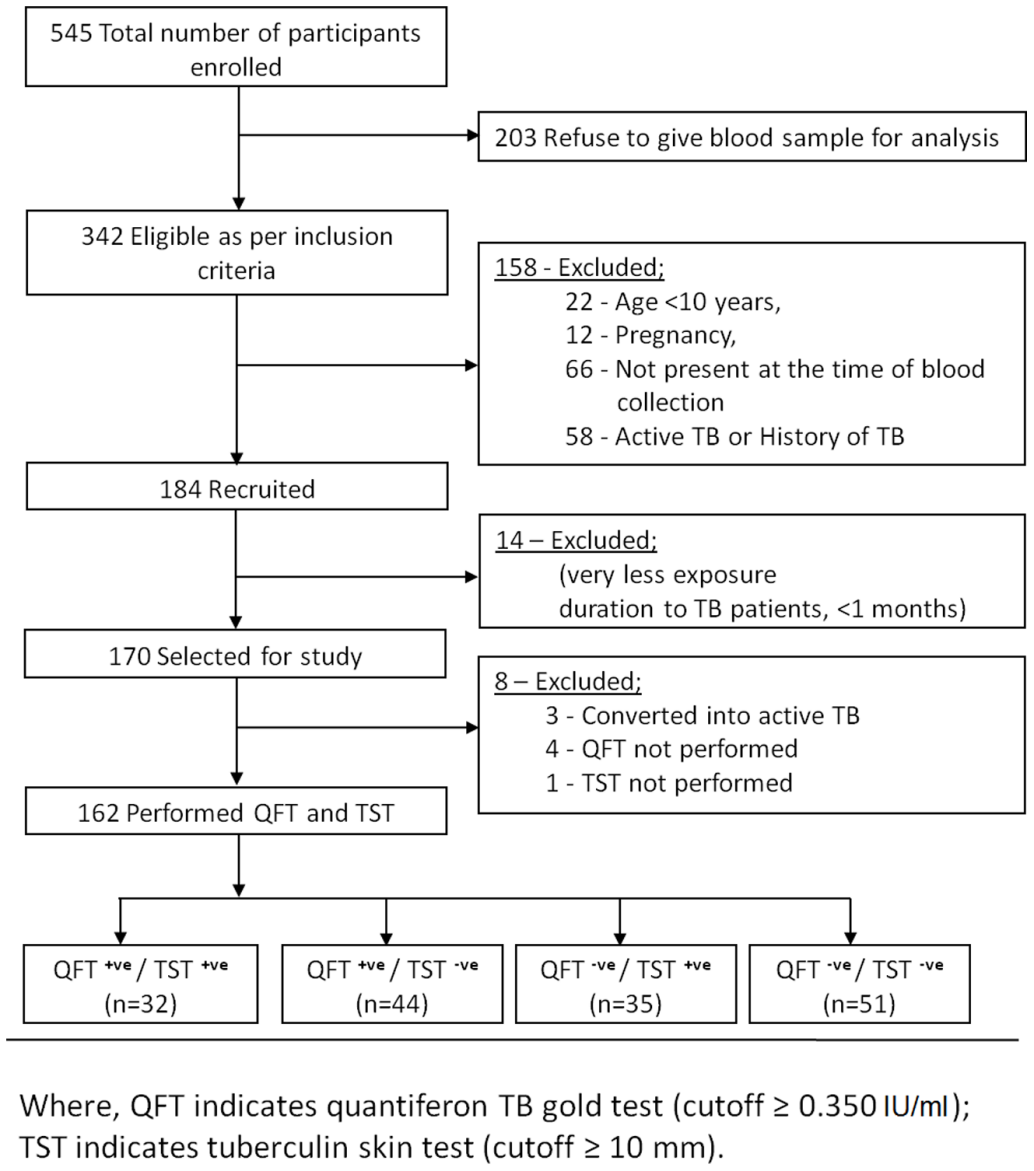
Study flow diagram.

Out of 342 eligible exposed participants, 162 (47%) were eventually considered for the study as shown in the flow chart (Figure 1). General characteristics of the TB exposed cases are shown in Table 1. Approximately 72% of exposed participants were below the age of 40 years. This young age group involved all 31 students and 58 individuals from labour class, suggesting 76% of the exposed participants in contact with the outside world. The overall proportion of underweight individuals was much higher (∼54%). BCG vaccination was confirmed only in 67 participants. Majority, i.e. 112 (∼70%) of participants had moderate duration of exposure and 100 (∼62%) had indirect contact with the active TB patients.

**Table 1:** General characteristics of TB exposed cases (*n* = 162).

Characteristics	Levels	No. (%) of cases
Age (years)		
	*<18*	59 (36.41)
	*18–40*	58 (35.80)
	*>40*	45 (27.77)
Sex	(M/F)	58/104
Occupation		
	*Student*	31 (19.13)
	*House wife*	47 (29.01)
	*Labour*	84 (51.85)
Education	*Literate*	125 (77.16)
	*Illiterat*e	37 (22.8)
BMI (kg/m2)	*Normal*	58 (35.80)
	*Underweight*	88 (54.32)
	*Overweight*	16 (00.09)
Symptoms		
	*Weight loss*	23 (14.19)
	*Cough with expectorant*	52 (32.09)
	*Cough without expectorant*	15 (09.25)
	*Fever*	39 (24.07)
	*Stomach pain*	21 (12.96)
	*Chest pain*	20 (12.34)
Behavioural factors		
	*Smoking*	4 (02.46)
	*Alcohol consumption*	2 (01.23)
	*Tobacco chewing*	14 (08.64)
BCG vaccinated†		
	*Yes*	67 (57.26)
	*No*	50 (42.74)
Duration of exposure		
	*Moderate*	112 (69.13)
	*High*	50 (30.87)
Contact		
	*Indirect*	100 (61.72)
	*Direct*	62 (38.28)

† Out of 117.

### Agreement between QFT-G and TST

Table 2 summarizes the agreement/concordance between QFT-G assay and TST results for varying cut-offs of TST and a fixed QFT-G cut-off of 0.35 IU/ml. Percent agreement and Cohen's Kappa statistic were obtained for each combination of cut-offs. For a cut-off point of 5 mm, out of 162, 71 (43.8%) participants were positive, while 16 (9.87%) were negative by both the tests. Remaining 75 (46.3%) were positive by either of the tests, giving an overall test agreement of 53.7%. The kappa statistic obtained was 0.098 (95% CI: 0.00–0.20), indicating a poor association between the tests. On similar lines, the agreements for 10 mm and 15 mm TST cut-off points were 52.47% and 55.56% respectively. Although the concordance was higher for TST cut-offs of 5 mm and 15 mm when compared to 10 mm it differed insignificantly as confirmed using *z*-test for proportion through pair wise comparisons. We referred to TST cut-point of at least 10 mm for deciding test positivity, as reported in different studies [11], for the further analysis.

**Table 2:** Correlation between the QFT-G and TST assays in exposed population (*n* = 162).

QFT result	Mantoux result					
	≥5 mm		≥10 mm		≥15 mm	
	Positive	Negative	Positive	Negative	Positive	Negative
Positive	71	7	34	44	19	59
Negative	68	16	33	51	13	71
*Agreement*	53.7%		52.47%		55.56%	
*Cohen's Kappa*	0.098		0.043		0.091	
*95% CI*	[−0.008, 0.204]		[−0.113, 0.199]		[−0.037, 0.219]	
*P-value*	0.033		0.289		0.078	

### Relevance of risk factors with test outcomes

- **QFT-G.** Following the cut-off criterion of ≥0.35 IU/mL, 78 (48%; 95% CI: 40–56%) out of 162 participants were positive for QFT-G. Table 3 shows the relevance of risk factors with QFT-G assay positivity. Bivariate analysis suggested statistically significant association of odd ratio (OR) of age, occupation, BCG vaccination, duration of exposure and type of contact with QFT-G positive outcome (*P*<0.05). These factors were considered together in the multivariate logistic regression model, except BCG vaccination due to its missing values. A stepwise backward elimination procedure using Wald's criteria with F-to-enter threshold of 0.05 and F-to-remove threshold of 0.1 was used to arrive at a parsimonious model. The final model included age and duration of exposure as the most relevant risk factors associated with QFT positivity. The Hosmer and Lemeshow test was insignificant with *P*-value of 0.603 (*P*>0.05) indicating a good fit of the model, with 67.3% correct predictive capacity. In the final model, the odds ratio for age revealed that odds of LTBI positivity by QFT-G increased significantly for age group of 18–40 years (OR: 4.75; 95% CI: 1.94–11.62) and for age beyond 40 years (OR: 6.31; 95% CI: 2.43–16.34) as compared to reference group <18 years. Both these odds ratio increased by about 1.71 times of the respective unadjusted estimates. Another risk factor duration of exposure, indicated highly significant (*P*<0.0001) increase in the odds ratio (OR: 6.28; 95% CI: 2.68–14.73) when the exposure duration is high as compared to moderate. The odds ratio for duration of exposure in the parsimonious model also increased 1.7 times that of unadjusted estimate.
- **Mantoux test (TST).** On similar lines, the analysis was performed with the TST as outcome variable for the same set of 162 participants. A cut-off criterion of ≥10 mm, resulted into 67 (41%; 95% CI: 34–50) participants with LTBI positive. This criterion was used to understand the relevance of risk factors with TST positive outcome (Table 4). Bivariate analysis suggested BMI and duration of exposure as statistically relevant risk factors of LTBI. A multivariable logistic regression with these two predictors was performed to arrive at a parsimonious model following the same variable selection criteria as above. The final model also included these two predictors with a Hosmer and Lemeshow test significance of 0.984 (*P*>0.05) indicating a good fit, and providing 65.4% predictions accuracy. Table 4 reveals that the increase in the odds of TST positivity for participants with normal BMI was 2.1 (95% CI: 0.99–4.46) times more than those in the underweight category. This association was contradictory to that obtained for QFT outcome. For overweight individuals, the odds ratio was 0.42 (95% CI: 0.10–1.75) compared to underweight category, which matched with QFT-G results, although statistically insignificant. Duration of exposure emerged out as the other significant risk factor for TST positive outcome. The odds of positive outcome increased 3.33 (95%: 1.61–6.88) times for higher exposure as compared to moderate exposure. As compared to unadjusted estimates, the increase in the respective odds ratio for these two factors was 1.1 times. Thus, amongst risk factors, duration of exposure appeared to be the most relevant factor associated with LTBI positivity irrespective of the test. Moreover, age had important role in QFT-G positivity, while BMI had a role in TST positivity.

**Table 3:** Correlation between patient characteristics and QFT results obtained through bivariate and multivariable logistic regression analysis (*n* = 162).

Characteristic	Levels	QFT result (cut-off ≥0.35 IU/ml)		
		Positive cases/Total cases (%)	OR (95% CI)	
			Unadjusted‡	Adjusted†
Age (years)				
	*<18*	18/59 (30.50)	1.00	1.00
	*18–40*	32/58 (55.17)	2.77 (1.30–6.03)*	4.75 (1.94–11.62)**
	*>40*	28/45 (62.22)	3.68 (1.64–8.58)*	6.31 (2.43–16.34)**
Sex				
	*Male*	30/58 (51.72)	1.00	
	*Female*	48/104 (46.15)	0.80 (0.42–1.53)	
Occupation				
	*Student*	7/31 (22.58)	1.00	
	*Housewife*	28/47 (59.57)	4.88 (1.81–14.63)*	
	*Labour*	43/84 (51.19)	3.51 (1.41–9.76)*	
Education				
	*Illiterate*	23/40 (57.50)	1.00	
	*Literate*	55/122 (45.08)	0.61 (0.29–1.25)	
BMI (kg/m2)				
	*Underweight*	43/88 (48.86)	1.00	
	*Normal*	28/58 (48.27)	0.97 (0.50–1.90)	
	*Overweight*	7/16 (43.75)	0.82 (0.26–2.43)	
BCG+				
	*No*	31/50 (62.00)	1.00	
	*Yes*	25/67 (37.31)	0.37 (0.17–0.78)*	
Duration of exposure				
	*Moderate*	43/112 (38.39)	1.00	1.00
	*High*	35/50 (70.00)	3.69 (1.83–7.76)*	6.28 (2.68–14.73)**
Contact				
	*Indirect*	41/100 (41.00)	1.00	
	*Direct*	37/62 (59.67)	2.11 (1.11–4.08)*	

*P<0.05;

**P<0.0001;

‡ Bivariate analysis;

† Parsimonious multivariate logistic regression model;

+ *n* = 117 (45 unknown).

**Table 4:** Correlation between patient characteristics and Mantoux (TST) results obtained through bivariate and multivariable logistic regression analysis (*n* = 162).

Characteristic	Levels	Mantoux test (cut-point ≥10 mm)		
		Positive case/Total cases (%)	OR (95% CI)	
			Unadjusted‡	Adjusted†
Age (years)				
	*<18*	24/59 (40.67)	1.00	
	*18–40*	24/58 (41.38)	1.03 (0.49–2.16)	
	*>40*	19/45 (42.22)	1.06 (0.48–2.35)	
Sex				
	*Male*	25/58 (43.10)	1.00	
	*Female*	42/104 (40.38)	0.89 (0.46–1.73)	
Occupation				
	*Student*	9/31 (29.03)	1.00	
	*Housewife*	17/47 (36.17)	1.37 (0.52–3.80)	
	*Labour*	41/84 (48.81)	2.29 (0.96–5.85)	
Education				
	*Illiterate*	13/40 (32.50)	1.00	
	*Literate*	56/122 (45.90)	1.89 (0.89–4.22)	
BMI (kg/m2)				
	*Underweight*	33/88 (37.50)	1.00	1.00
	*Normal*	31/58 (53.44)	1.90 (0.97–3.76)*	2.10 (0.99–4.46)*
	*Overweight*	3/16 (18.75)	0.40 (0.08–1.38)	0.42 (0.10–1.75)
BCG+				
	*No*	22/50 (44.00)	1.00	
	*Yes*	17/67 (25.37)	0.44 (0.19–0.95)	
Duration of exposure				
	*Moderate*	37/112 (33.03)	1.00	1.00
	*High*	30/50 (60.00)	3.01 (1.51–6.09)*	3.33 (1.61–6.88)*
Contact				
	*Indirect*	38/100 (38.00)	1	
	*Direct*	29/62 (46.77)	1.43 (0.75–2.73)	

*P<0.05;

‡ Bivariate analysis;

† Parsimonious multivariate logistic regression model;

+ *n* = 117 (45 unknown).

### Association of risk factors with patterns of discordance

The association between risk factors and the patterns of discordance of tests was studied through multinomial multivariable logistic regression. Four patterns of concordance and discordance i.e., concordance negative and positive, and two discordant types QFT-G −/TST+ and QFT-G +/TST− were considered as outcomes, and the factors were treated as predictors. Negative concordance was considered as reference level and the risks associated with each factor for the other three outcomes were obtained in terms of adjusted odds ratio as shown in Table 5. Education was significantly associated with QFT-G−/TST + discordance (OR: 6.45; 95%CI: 1.37–30.30). Also, normal BMI category compared to underweight showed significant relevance (OR: 3.32; 95% CI: 1.26–8.72) with QFT-G−/TST + discordance. This observation was consistent with that of TST outcome in Table 4. For QFT-G +/TST – discordance, age category 18–40 years showed significant association (OR: 5.46; 95% CI: 1.91–15.62) as compared to reference level of <18 years. Housewives and Labour class also showed significant relevance with this discordance with odds ratio 5.14 (95% CI: 1.40–18.58) and 5.68 (95% CI: 1.64–19.63) respectively compared to student level. No other factor showed significant association with this discordance. For concordant positive outcome, occupation and duration of exposure showed significant relevance. Housewife and labor class had significantly increase odds ratios of 5.15 (95% CI: 1.21–21.82) and 6.00 (95% CI: 1.00–35.99) as compared to student category. Further, higher duration of exposure was significantly associated with concordant positive result (OR: 11.82; 95% CI: 4.19–33.30). Moreover, direct contact also showed significant association with concordant positive result with OR 3.23 (95% CI: 1.31–7.97). These results corroborated with that of QFT-G outcome in Table 3. Model fitness was evaluated each time and except sex, the model chi-square based on log-likelihood indicated statistical significance, suggesting significant deviation of predictor coefficients from zero. In other words, these predictors had significant association with the discordant/concordant positive outcome compared to reference concordant negative.

**Table 5:** Multinomial univariate logistic regression to determine the correlation of risk factors with concordant and discordant test results.

Characteristics	Levels	Odds ratio (95% CI)		
		QFT−/TST+ (*n* = 35)	QFT+/TST− (*n* = 44)	QFT+/TST+ (*n* = 32)
Age (years)	*<18*	1.00	1.00	1.00
	*18–40*	1.91 (0.71–5.26)	5.46 (1.91–15.62)*	2.28 (0.77–6.75)
	*>40*	1.83 (0.52–6.45)	1.18 (0.42–3.35)	0.72 (0.23–2.22)
Gender	*Male*	1.00	1.00	1.00
	*Female*	0.80 (0.32–2.01)	0.73 (0.31–1.69)	0.74 (0.29–1.83)
Occupation	*Student*	1.00	1.00	1.00
	*House wife*	1.07 (0.27–4.25)	5.14 (1.40–18.58)*	5.15 (1.21–21.82)*
	*Labour*	3.47 (1.14–10.53)	5.68 (1.64–19.63)*	6.00 (1.51–23.79)*
Education	*Illiterate*	1.00	1.00	1.00
	*Literate*	6.45 (1.37–30.30)*	0.99 (0.41–2.41)	1.00 (0.38–2.59)
BMI (kg/m^2^)	*Underweight*	1.00	1.00	1.00
	*Normal*	3.32 (1.26–8.72)*	1.61 (0.64–4.08)	1.77 (0.67–4.66)
	*Overweight*	0.28 (0.03–2.43)	0.81 (0.23–2.78)	0.41 (0.08–2.12)
Duration	*Low*	1.00	1.00	1.00
	*High*	0.50 (0.14–1.73)	0.93 (0.35–2.52)	11.82 (4.19–33.30)**
Contact	*Indirect*	1.00	1.00	1.00
	*Direct*	0.64 (0.24–1.72)	1.14 (0.49–2.66)	3.23 (1.31–7.97)*

*P<0.05;

**P<0.0001;

QFT−/TST – as reference level.

Table 6 shows QFT-G and TST analysis in follow up participants along with baseline data. Out of 162 participants, follow up was available for only 8 participants (5%). Participants recruited were mostly females (60%) in underweight category. In recruited 8 participants, only 1 participant showed sero conversion from concordance negative to concordance positive. The particular participant was reported in the age group of 40–60 years, with moderate duration of exposure and reported direct contact with TB patient. On basis of QFT-G, there were 5 participants that showed QFT-G positivity in initial and follow-up studies. In the same group, 4 participants showed higher QFT-G levels in follow-up compared to initial QFT-G levels. These participants were in direct contact with TB patients, while **1** participant showed indirect exposure. Out of 5, 3 participants were among those which had high duration of exposure with TB patients, while 2 showed moderate exposure. Also out of 5, only 1 had taken BCG, while there were 2 participants with no prior BCG vaccination. BCG status was not known in remaining 2 cases. 2 participants were negative in initial and follow up studies. Based on TST, 1 participant showed sero conversion from TST negative to TST positive in follow-up study, while there were 3 cases reported which remained TST positive in follow-up as well. Follow-up was not available in 2 participants which were QFT-G positive in both studies. 1 participant remained TST negative in both initial and follow up studies

**Table 6:** QFT and TST assay in initial and Follow studies and their correlation with Baseline characteristics.

Case Details	Age (in years)	Sex	Occupation	Education	Baseline BMI	BCG	Exposure Duration	Contact type	QFT values	QFT Result	Follow up QFT values	Follow up QFT Result	TST values	TST Result	Follow up TST values	Follow up TST Result
Case-1* ¥	40–60	Male	Labour	literate	Normal	NA	Moderate	Direct	0	Negative	1.46	Positive	9 mm	Negative	10 mm	Positive
Case-2# ¥	<18	Male	Student	literate	Underweight	NA	Moderate	Indirect	12.63	Positive	18.27	Positive	5 mm	Negative	11 mm	Positive
Case-3$ ¶	18–40	Female	House wife	literate	Underweight	NA	Moderate	Indirect	0.27	Negative	0.04	Negative	24 mm	Positive	11 mm	Positive
Case-4# ¥	18–40	Female	House wife	literate	Underweight	Yes	Moderate	Direct	6.35	Positive	15.84	Positive	9 mm	Negative	11 mm	Positive
Case-5$ ¶	<18	Female	Student	literate	Underweight	Yes	Moderate	Indirect	0.3	Negative	0.14	Negative	10 mm	Positive	11 mm	Positive
Case-6# Φ	18–40	Female	House wife	literate	Underweight	NA	High	Direct	1.46	Positive	2.95	Positive	11 mm	Positive	ND	ND
Case-7# ¶	40–60	Male	Labour	illiterate	Underweight	NO	High	Direct	9.28	Positive	3.22	Positive	22 mm	Positive	18 mm	Positive
Case-8# Φ	<18	Female	Student	literate	Underweight	NO	High	Direct	1.9	Positive	14.88	Positive	14 mm	Positive	ND	ND

NA - Not Available.

ND - Not Done.

*Conversion of QFT Test into positive after follow up.

# QFT Test still remains positive after follow up.

$ QFT Test still remains negative after follow up.

¥ Conversion of TST Test into positive after follow up.

¶ TST Test still remains positive after follow up.

Φ TST was not done in follow up cases.

## Discussion

In India, limited measures are undertaken for LTBI diagnosis, despite of its vast prevalence in high TB endemic regions. In the present study we investigated utility of two routinely used diagnostic tests for LTBI to estimate its prevalence in high TB endemic settings of Nagpur, India. To our knowledge, the present study is among the first to be reported with the participants from specific locality in Nagpur.

Based on the results, our study demonstrated high prevalence of LTBI in participants, which is in accordance with the estimates from other studies in developing countries [11], [12], [13]. With TST, we reported an estimate of 42% prevalence; while, with QFT-G the prevalence was 48%. Besides, the combination of these tests (either of test positive) showed a prevalence of around 69% among the participants. Risk factors identified in current study have been shown to be important in several other studies [4], [14], [15], [16].

On bivariate analysis, significant association (OR) of age, occupation, BCG vaccination, duration of exposure and type of contact with QFT positivity was observed. When these associated risk factors (except BCG) were driven in the multivariable model, age and duration of exposure emerged as important risk factors for QFT-G positivity. Age has been regarded as a strong predictor for QFT-G positivity. The observation is well supported by a study conducted on Japanese health care worker in which age along with history of working in a TB ward, were significantly associated with QFT-G positivity [17]. However no significant impact of age was observed with TST positivity. Major reason for this could be less specificity and false positivity associated with TST in high TB endemic zones with age due to high environmental exposure [18].

BMI was found to be major confounding factor on observed TST positvity suggesting the association of nutrition as a risk factor with the development of LTBI in exposed population. There is substantial evidence that suggest malnutrition as a possible determinant of TB [19], [20], however the association of same with TST positivity in exposed population seems interesting. There are various contradictions associated with impact of malnutrition on TST positivity. Reports suggest that antigen-specific immunosuppression (anergy) caused due to malnutrition may cause false negative TST [18]. In our study we found similar impact of BMI on TST where risk of getting positive TST was almost doubled in normal as compared to underweight participants.

Duration of exposure was identified as major risk factor associated with both the evaluated tests. The risk of LTBI is dependent on many factors in which exogenous factors play a very important role. High duration of exposure with TB patients, the degree of infectiousness of the TB patient along with shared environment in which the contact takes place are important risk factors modulating LTBI which also have been described in many studies [21], [22], [23], [24]. In our study, participants recruited belonged to families with high household crowding index with improper ventilation existing in most of the houses. Overcrowding exacerbate the risk of exposure due to poor ventilation hence affecting the health status by making participants more prone towards TB. A study in Cape Town reported that individuals with high MTB exposure were more likely to have a positive impact on test [25]. In another study in Senegal, the proximity to index case were strongly associated with the positivity of the test [5] indicating that the duration of exposure is the key factor in determining the risk of developing TB in exposed population.

Along with duration of exposure, type of contact with TB patient is also major confounding factor that determines outcome of QFT-G and TST [11], [14]. In our study, through bivariate analysis, we found a significant impact of contact type on observed positivity of both the tests. However, due to high co-linearity associated with both duration of exposure and contact type, the latter was excluded in multivariate analysis.

BCG vaccination was reported to have positive impact on TST [26]. However, in the present study we found a significant association of BCG with both tests, suggesting a probability of LTBI diagnosis higher in non BCG vaccinated participants. Even though we found a protective role of BCG in the study, no conclusion can be obtained from our findings as about 45 participants were unsure of their BCG vaccination status, hence they were excluded from the model.

An agreement of 52.47% was reported between the two tests in the study population, consistent with the other studies done in high TB burden settings [11], [25], [13]. Discordance between TST and QFT-G in terms of LTBI diagnosis has been reported in low, intermediate and high TB burden settings, suggesting that either of the tests may be working better than other in present settings [8], [14], [27]. Since QFT-G test are particularly designed to overcome most of the limitation of TST and are regarded to be more specific than TST, perfect agreement between the two tests was difficult to obtain [28] in TB endemic regions. Our study contradicts with the investigation done by Pai *et al.* [11] in health care workers, showing a high agreement between the two tests [15]. Main reasons for the disagreement may be due to the differences in non BCG vaccinated study population and study settings. As explained earlier and also reported in several other studies, BCG has significant impact on TST positivity [28].

As mentioned elsewhere that QFT-G assay has higher specificity and less cross reactivity than TST suggesting that QFT-G may be more helpful in screening LTBI in the present setting. However, our results along with other evidences suggest that both assays have advantages and limitations, therefore both have useful roles depending on the factors unique to each setting. Hence, the combination of QFT-G and TST will be considerably useful in LTBI diagnosis in household contacts of TB patients in high TB endemic regions.

Although the present study enlightens important findings and suggests the possibility of association of duration of exposure with the development of LTBI, the study has some limitations. The major limitations of our study include small sample size and unknown BCG vaccination status of enrolled participants. To further evaluate the diagnostic utility of QFT-G and TST, a follow up study was planned. However in Indian context various constraints exist in performing follow up studies. Unawareness, illiteracy, community ethics and mere fear of having TB disease excludes many participants from coming for follow up. However, after brief counseling we were able to collect follow up samples from 8 contacts in three years.

Based on results of follow up we reported only 1 participant with sero conversion from QFT-G/TST negative to QFT-G/TST positive, while 5 QFT-G positive participants remained positive in follow up as well. Interestingly out of 5, 4 participants showed abrupt increase with respect to their QFT-G values. With TST, we reported conversion of 1 participant, which was QFT-G positive while 3 participants remained positive in both initial and follow up studies. All participants showing conversion or higher positivity in both initial and follow up studies were mostly having direct contact with TB patients with moderate and high duration of exposure and were not given ATT. All participants later on developed clinical signs and symptoms related to TB. Based on the baseline data, all the mentioned factors may play an important role in conversion. Factors like duration of exposure and type of contact are particularly important risk factors and therefore may be assessed in follow up studies for observing conversion to active TB.

Even though in our follow up studies, we were able to recruit only eight participants, but still it highlighted some important observations, suggesting that exposed QFT-G and TST positive participants which failed to come for follow up may have a higher risk of developing active TB in their later stages of life. Their conversion is well supported by LTBI biomarkers like QFT-G and TST.

In conclusion, our study showed high prevalence of LTBI in participants living in high crowding index areas in TB endemic regions. Advantages and limitations associated with both the tests suggested that combinatorial approach may be more useful in detecting LTBI. However, further prospective studies should be planned for assessing both QFT-G and TST along with their correlation with follow up cases for LTBI diagnosis in such TB endemic population in India.

## Supporting Information

Table S1 **Association between patient characteristics and QFT/TST results obtained through bivariate and multivariable logistic regression analysis for confirmed BCG status sample (** ***n*** **= 117).**
(DOC)

File S1 **Subgroup analysis in BCG vaccinated and unvaccinated individuals in studied population.**
(DOC)

File S2 Equation for parsimonious multivariate logistic regression model for a) QFT and b) TST as outcome.
(DOC)
