# Supplementary material for: Latent TB Infection Diagnosis in Population Exposed to TB Subjects in Close and Poor Ventilated High TB Endemic Zone in India
Source: PLoS One. 2014 Mar 10;9(3):e89524. doi: 10.1371/journal.pone.0089524 (PMC3948673; doi:10.1371/journal.pone.0089524)
Supplement: Table S1 — Association between patient characteristics and QFT/TST results obtained through bivariate and multivariable logistic regression analysis for confirmed BCG status sample ( n = 117). (DOC) [file pone.0089524.s001.doc]

**Table S2:** Association between patient characteristics and QFT / TST results obtained through bivariate and multivariable logistic regression analysis for confirmed BCG status sample (*n*=117)

|  |  | QFT | | |  | Mantoux test | | |
| --- | --- | --- | --- | --- | --- | --- | --- | --- |
| Characteristics |  | Positive case / | OR (95% CI) | |  | Positive case / | OR (95% CI) | |
|  |  | Total cases (%) | Unadjusted | Adjusted† |  | Total cases (%) | Unadjusted | Adjusted† |
| Age (years) |  |  |  |  |  |  |  |  |
| *< 18* |  | 13/44 | 1 |  |  | 13/44 | 1 |  |
| *18 - 40* |  | 22/38 | 3.21 (1.29 - 8.27) |  |  | 11/38 | 0.97 (0.36 - 2.55) |  |
| *> 40* |  | 21/35 | 3.49 (1.38 - 9.24) |  |  | 15/35 | 1.77 (0.69 - 4.61) |  |
| Sex |  |  |  |  |  |  |  |  |
| *Male* |  | 22/39 | 1 |  |  | 13/39 | 1 |  |
| *Female* |  | 34/78 | 0.60 (0.27 - 1.31) |  |  | 26/78 | 0.99 (0.44 - 2.31) |  |
| Occupation |  |  |  |  |  |  |  |  |
| *Student* |  | 7/30 | 1 | 1 |  | 8/30 | 1 |  |
| *Housewife* |  | 23/40 | 4.29 (1.53 - 13.23) | **7.83 (2.30 - 26.65)** |  | 12/40 | 1.17 (0.41 - 3.51) |  |
| *Service* |  | 26/47 | 3.94 (1.45 - 11.80) | **6.80 (2.08 - 22.28)** |  | 19/47 | 1.83 (0.68 - 5.25) |  |
| Education |  |  |  |  |  |  |  |  |
| *Illiterate* |  | 21/39 | 1 |  |  | 10/39 | 1 |  |
| *Literate* |  | 35/78 | 0.70 (0.32 - 1.52) |  |  | 29/78 | 1.69 (0.73 - 4.16) |  |
| BMI (kg/m^2^) |  |  |  |  |  |  |  |  |
| *Underweight* |  | 27/59 | 1 |  |  | 16/59 | 1 | 1 |
| *Normal* |  | 24/45 | 1.34 (0.61 - 2.97) |  |  | 22/45 | 2.54 (1.12 - 5.88) | **3.16 (1.31 - 7.61)** |
| *Overweight* |  | 5/13 | 0.75 (0.19 - 2.57) |  |  | 1/13 | 0.25 (0.009-1.48) | 0.29 (0.034-2.52) |
| BCG |  |  |  |  |  |  |  |  |
| *No* |  | 31/50 | 1 |  |  | 22/50 | 1 |  |
| *Yes* |  | 25/67 | 0.56 (0.27 - 1.13) |  |  | 17/67 | 0.44 (0.19 - 0.95) |  |
| Duration of exposure |  |  |  |  |  |  |  |  |
| *Low* |  | 30/79 | 1 | 1 |  | 20/79 | 1 | 1 |
| *High* |  | 26/38 | 3.47 (1.55 - 8.18) | **5.87 (2.18 - 15.84)** |  | 19/38 | 2.91 (1.29 - 6.69) | **3.34 (1.38 - 8.07)** |
| Contact |  |  |  |  |  |  |  |  |
| *Indirect* |  | 28/70 | 1 |  |  | 22/70 | 1 |  |
| *Direct* |  | 28/47 | 2.18 (1.03 - 4.73) |  |  | 17/47 | 1.23 (0.56 - 2.71) |  |

† Parsimonious multivariate logistic regression model
