# Supplementary material for: Latent TB Infection Diagnosis in Population Exposed to TB Subjects in Close and Poor Ventilated High TB Endemic Zone in India
Source: PLoS One. 2014 Mar 10;9(3):e89524. doi: 10.1371/journal.pone.0089524 (PMC3948673; doi:10.1371/journal.pone.0089524)
Supplement: File S1 — Subgroup analysis in BCG vaccinated and unvaccinated individuals in studied population. (DOC) [file pone.0089524.s002.doc]

**S1:** Subgroup analysis in BCG vaccinated and unvaccinated individuals in studied population

A subgroup analysis was performed for confirmed BCG vaccinated or not vaccinated individuals (*n*=117). Regarding test concordance, 61% (72/117) agreement was obtained for TST cut-point of 10mm, followed by 56% (66/117) for 5mm and 55% (65/117) for 15 mm. Thus for subgroup, the change in the concordance levels for 5 mm and 15 mm remained almost the same, while for 10 mm, there was substantial increase in the concordance from 52.47% although insignificant (*P*=0.166). The association of covariates was studied by including age, sex, occupation, education, BMI, BCG status, duration of exposure and contact in the logistic regression model with either QFT-G or TST positive as outcome. In the bivariate analysis with 117 observations, BCG vaccination had significantly reduced odds (OR: 0.37; 95% CI: 0.17 – 0.78) in favor of QFT-G positivity (Table 2). However, in the final multivariate model for the subset, the likelihood of QFT-G positivity although reduced (OR: 0.61; 95% CI: 0.25 – 1.48), the finding was statistically insignificant (*P*=0.278). A parsimonious model derived through Wald’s backward elimination resulted into occupation and duration of exposure as the two key risk factors of QFT-G positivity. Similar analysis for TST outcome revealed BMI and duration of exposure as the most relevant predictors of TST positivity [Supplementary table]. Thus, for TST, the results of subgroup with BCG as one of the covariate were consistent with that of the original model without BCG (*n*=162). Duration of exposure was once again confirmed as the important risk factor of test positivity.
