# Supplementary material for: Latent TB Infection Diagnosis in Population Exposed to TB Subjects in Close and Poor Ventilated High TB Endemic Zone in India
Source: PLoS One. 2014 Mar 10;9(3):e89524. doi: 10.1371/journal.pone.0089524 (PMC3948673; doi:10.1371/journal.pone.0089524)
Supplement: File S2 — Equation for parsimonious multivariate logistic regression model for a) QFT and b) TST as outcome. (DOC) [file pone.0089524.s003.doc]

**S3:** Equation for parsimonious multivariate logistic regression model for a) QFT and b) TST as outcome

A) **QFT as Outcome**

f(x)=1.842age(1)+1.559age(2)+1.838duration(1)-1.722

where

the variable coding for age is:

code

age (1) (2)

<18 0 0

>40 1 0

18-40 0 1

The variable coding for duration is:

Duration Low 0

High 1

B) **TST as outcome**

g(x)=0.792bmi(1)-0.795bmi(2)+1.176duration(1)-0.957

The variable coding for bmi is:

code

1 2

underweight 0 0

normal 1 0

overweight 0 1

The variable coding for duration is:

Duration Low 0

High 1
